# Estimating the potential impact of behavioral public health interventions nationally while maintaining agreement with global patterns on relative risks

**DOI:** 10.1371/journal.pone.0232951

**Published:** 2020-05-13

**Authors:** Ozden Gur Ali, Angi Nazih Ghanem, Bedirhan Ustun

**Affiliations:** 1 College of Administrative Sciences and Economics, Koc University, Istanbul, Turkey; 2 College of Engineering, Koc University, Istanbul, Turkey; 3 School of Medicine, Koc University, Istanbul, Turkey; Sciensano, BELGIUM

## Abstract

**Objective:**

This paper introduces a novel method to evaluate the local impact of behavioral scenarios on disease prevalence and burden with representative individual level data while ensuring that the model is in agreement with the qualitative patterns of global relative risk (RR) estimates. The method is used to estimate the impact of behavioral scenarios on the burden of disease due to ischemic heart disease (IHD) and diabetes in the Turkish adult population.

**Methods:**

Disease specific Hierarchical Bayes (HB) models estimate the individual disease probability as a function of behaviors, demographics, socio-economics and other controls, where constraints are specified based on the global RR estimates. The simulator combines the counterfactual disease probability estimates with disability adjusted life year (DALY)-per-prevalent-case estimates and rolls up to the targeted population level, thus reflecting the local joint distribution of exposures. The Global Burden of Disease (GBD) 2016 study meta-analysis results guide the analysis of the Turkish National Health Surveys (2008 to 2016) that contain more than 90 thousand observations.

**Findings:**

The proposed Qualitative Informative HB models do not sacrifice predictive accuracy versus benchmarks (logistic regression and HB models with non-informative and numerical informative priors) while agreeing with the global patterns. In the Turkish adult population, Increasing Physical Activity reduces the DALYs substantially for both IHD by 8.6% (6.4% 11.2%), and Diabetes by 8.1% (5.8% 10.6%), (90% uncertainty intervals). Eliminating Smoking and Second-hand Smoke predominantly decreases the IHD burden 13.1% (10.4% 15.8%) versus Diabetes 2.8% (1.1% 4.6%). Increasing Fruit and Vegetable Consumption, on the other hand, reduces IHD DALYs by 4.1% (2.8% 5.4%) while not improving the Diabetes burden 0.1% (0% 0.1%).

**Conclusion:**

While the national RR estimates are in qualitative agreement with the global patterns, the scenario impact estimates are markedly different than the attributable risk estimates from the GBD analysis and allow evaluation of practical scenarios with multiple behaviors.

## Introduction

Non-communicable diseases account for the majority of the global burden of disease. 72% of all deaths were estimated to be due to non-communicative diseases in 2016 [1]. Changing behaviors, such as exercise and healthy diet has the potential to decrease, or slow the increase of public burden [2,3]. According to the World Health Organization (WHO), as of 2017, 161 out of the 194 countries had operational policy/ strategy/ action plan to decrease tobacco use, and 100 had implemented physical activity public awareness programs [4]. Beyond global measures, national and local evidence is needed [5]. Quantifying the potential impact of a behavior change in the local population enables policy makers to use resources more efficiently. Clearly, the policy maker’s decision as to which intervention (if any) to pursue also depends on the cost and likelihood of the potential interventions to achieve the intended behavior change.

Global organizations such as the WHO and Institute for Health Metrics and Evaluation (IHME) produce annual reports summarizing the impact of a large number of risk factors and causes of death [6,7]. For example, the GBD tool provides age, gender, year and location specific disability adjusted life years (DALY) and percent attributable burden to risk factors by cause of death or injury (https://vizhub.healthdata.org/gbd-compare/). These results are based on GBD studies that combine clinical and field evidence from thousands of sources, and smooth estimates across age, time and locations and to reconcile incidence, prevalence and mortality estimates with a consistent methodology. On the other hand, GBD 2016 [1] assumes that the global relative risk (RR) estimates are generalizable across populations, while acknowledging that the “RR due to low education for 40-year-old men would be different in Norway than in Kenya” with plans to quantify the differences.

The RR is combined with an estimate of the local exposure to the risk factor and DALY for the outcome to calculate the attributable burden of disease due to the risk factor, compared with a counterfactual risk exposure level [8,9]. Several studies on smoking [9,10], diet [11,12], and physical activity [13] use a similar methodology.

In order to obtain a more specific estimate for the impact of a behavior change scenario, some studies estimate the local risk exposure with local representative data (including national health surveys and observational studies), while using relative risk estimates from the literature, e.g. [14–16], for behavioral risks such as second hand smoke or diet. Others point out that most studies in the literature estimate RR with one single risk factor in isolation and advocate estimating the impact of risk factors jointly on local individual level data [e.g., 17].

Meta-analyses reflect the consensus in the academic community on causal effects, important determinants, direction and monotonicity of impact. On the other hand, local data allows estimation of the relative risks and exposures directly. Further, individual level data allows controlling for demographics, other behaviors and socioeconomic factors in the estimation of the relative risks; as well as in the calculation of the effect of multiple risk factors without double counting.

In this paper we offer a method to combine these benefits to evaluate the local impact of behavior change scenarios on disease prevalence and DALYs. The new method guides the estimation of disease probability models in a Hierarchical Bayes framework with the expected qualitative patterns regarding the direction and monotonicity of the effects based on GBD meta-analysis results. These models are combined with the individual level representative data to simulate scenarios on multiple behaviors and provide estimates of impact with uncertainty intervals.

The method offers the following benefits beyond the attributable burden estimates from GBD: a) incorporates the local relative risk rather than the global average, b) uses the local risk exposure, rather than the projections made based on exposure in other age/ gender/ country groups, c) can evaluate scenarios with multiple behaviors (risk factors), d) can evaluate scenario impact for targeted interventions at population segments defined by any observed characteristics, going beyond age and gender. Beyond providing better estimates by using local individual level data and estimating risk factors jointly, the proposed methodology provides RR estimates have the expected signs and patterns based on the collective wisdom of researchers embodied in the GBD study results.

We use the proposed method to evaluate the impact of behavior change scenarios involving smoking, physical exercise and diet on prevalence and DALYs due to ischemic heart disease (IHD) and diabetes mellitus in the Turkish adult population. IHD is the largest contributor to the burden of disease in Turkey, accounting for 8.3%, while diabetes contributes 4% and is growing at a very high rate [https://vizhub.healthdata.org/gbd-compare/]. Promoting physical activity, healthy diet and reducing smoking are the top three goals in the strategic plan of the Ministry of Health. We use the biannual Health Surveys conducted by the Turkish Statistical Institute (TUIK) in years 2008 to 2016 with more than 90 thousand observations as the individual data source, along with the GBD 2016 results for the analysis.

## Materials and methods

The Human Subject Research was reviewed by the Koc University Ethics Committee with protocol number 2019.184.IRB3.114 on May 30, 2019 and considered exempt from ethical review.

### Method overview

**Fig 1:**
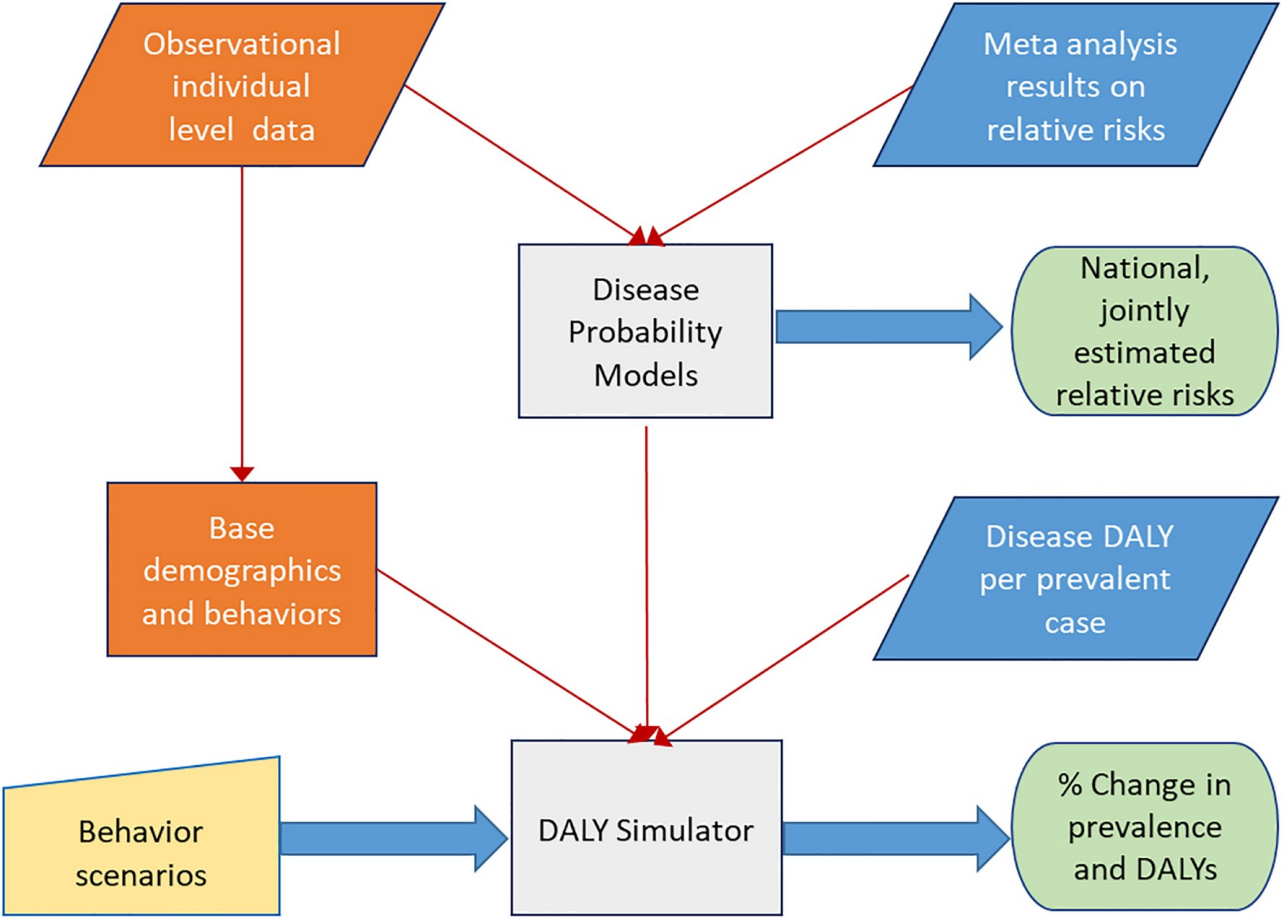
Overview of the proposed method. The two main components that are represented by the grey boxes. The diagonal boxes represent the data inputs, where the blue colored come from meta-analysis results and the orange colored are the observational individual level data. The user input is in yellow and the results are in green boxes.

Fig 1 provides an overview of the developed method, which has two main components: a) the Disease Probability Models predicting individual disease as a function of behaviors, demographics, socio-economics and other controls; b) the DALY Simulator that calculates the % change in DALYs and prevalence of the considered diseases for the given behavior scenario compared to the base case of behaviors. The most recent representative individual level data provides the base case for the joint distribution of behaviors, demographics and socioeconomic factors.

The global relative risk estimates from the meta-analysis results and the observational individual level data are used to estimate and evaluate the Disease Probability Models, whose coefficients can be interpreted as the natural log of the national relative risk estimates of behaviors. The DALY Simulator calculates the expected burden of disease before and after the behavior change at the individual level using predictions from the multiple disease probability models sharing the same set of inputs and the DALY per prevalent case (DPP) estimates. Rolling up the individual level burden provides the impact of changes in multiple behaviors without double-counting.

### Data sources

GBD studies present a comprehensive assessment of the worldwide health impact of disease, injury and risk factors, providing point and interval estimates at country-level; facilitating comparison of results by geography, time, age, gender and different health conditions [18–21]. From the meta-analysis results of the GBD 2016 study [7], we use the RR and the DPP estimates.

Specifically, we use the mean and 95% intervals of the RR estimates for the relevant risk factors by age and gender, (age, gender, smoking, second-hand smoking, physical activity, BMI) for Diabetes and IHD; (we do not use the fruit and vegetable consumption RR information since only the low consumption RR (less than 100g/day) is provided). We use the qualitative patterns that we extract from the quantitative estimates, i.e.; positive or negative, increasing or decreasing with age; monotone increasing or decreasing with exposure, to estimate the proposed Qualitative Informative HB model. We also use the qualitative patterns to evaluate whether the benchmark models are in agreement with these expected patterns. Please see first column of Table 1 for the extracted qualitative patterns: for example, the second row in the table indicates that the risk of both diseases, IHD and Diabetes, is higher for smokers vs non-smokers, inferred based on the RR estimates being greater than 1; while the second and third lines from the bottom indicate that the RR in monotonically decreasing with age for both males and females for IHD, while no age-specific estimate is provided for Diabetes. The first line in the table indicates that the for the multi-level risk factor physical activity, all other levels, i.e.; insufficiently active, active and very active, have lower levels of risk than the reference level inactive.

**Table 1:** Qualitative patterns extracted from the GBD 2016 relative risk estimates.

**Sign of the Effect (ln RR)**	IHD	Diabetes
Physical Activity (reference level Inactive)	negative	negative
Smoking (reference level Non-smoking)	positive	positive
Second-Hand Smoking (reference level Un-exposed)	positive	positive
BMI (reference level BMI<22.5)	positive	positive
**Shape of Effect**	IHD	Diabetes
Male with Age	increase	increase
Female with Age	increase	increase
Physical Activity with Activity Level	decrease	decrease
Physical Activity with Age	decrease	NA
Male Smoking with Age	decrease	NA
Female Smoking with Age	decrease	NA
BMI with Age	decrease	decrease

For one of the benchmark models, the Numerical Informative HB, we used the quantitative RR mean and interval estimates from GBD to specify the priors of the relevant parameters in model.

We calculate the disease, age and gender specific DPP estimates from the GBD 2016 study results reported in (http://ghdx.healthdata.org/gbd-results-tool), by dividing the estimates of disease DALYs by the number of prevalent cases in Turkey, for each age-gender group. DALY combines Years of Life Lost (YLL) due to premature mortality and the Years Lost due to Disability (YLD) for people living with the health condition or its consequences [22]. The DPP for IHD ranges by age and gender group from 0.24 to 0.83, and for Diabetes from 0.08 to 0.27, generally increasing with age–with the exception of male IHD values peaking in the 35–44 age group.

The nationally representative Health Surveys conducted biannually by the Turkish Statistical Institute (TUIK) 2008 to 2016 provide 93,528 observations of individuals 15 or older. More information about the surveys can be obtained from TUIK http://www.turkstat.gov.tr/PreHaberBultenleri.do?id=24573. We use all waves to train and evaluate the disease probability models, but use the latest wave (from year 2016) in the simulation to represent the base case scenario with current joint distribution of behaviors, demographics and socioeconomics. We use the following variables in addition to the self-reported presence of diseases, after transforming them to ensure compatibility with the relative risk groups from the meta-analysis.

*Age*: *a* = 1..7 for age buckets representing (15–24, 25–34, 35–44, 45–54, 55–64, 65–74, 75+); *Gender*: g = M,F male and female; *Physical_Activity*: *m* = 1..4 (“Inactive”, “Insufficiently active”, “Active”, “Highly Active”) defined as (<600 METs, 600–3999 METs, 4000–7999 METs and > = 8000 METs), which is calculated based on weighted activity minutes per week according to BRFSS users’ guide to physical activity [23]; *Smoke = 1* if a smoker, 0 otherwise; *SecSmoke* = 1 if exposed to secondhand smoke, 0 otherwise; *Alcohol*: *c* = 1..5 buckets representing alcohol consumption frequency (1 = “Never” and “Not anymore”, 2 = “Occasionally” and “Once a month”, 3 = “Couple a month”, 4 = “Once or twice a week”, 5 = “More than every other day”; *Vegetable*: v = 1..5, and *Fruit*: f = 1..5,where Vegetable and fruit consumption frequency buckets represent (1 =“none”, 2 =“less than once a week”, 3 =“1–3 times a week”, 4 =“4–6 times a week”, 5 = “one or more per day”); *Education*: years of education; *Income*: the income index adjusted for the effective household size (using the OECD methodology) and changing income levels over time; $BMI^{'}=max(0,\frac{BMI-22.5}{5})$; Trust: t = 1..4, Number of trusted individuals corresponding to (0, 1, 2, 3+) respectively; Region: r = 1..12, the 12 NUTS regions of Turkey; *Method* is a binary variable that controls for the change in survey methodology in 2014. Since 99% of the respondents level of alcohol consumption corresponds to less than the lowest level in the GBD analysis (12g pure alcohol/day) we do not use the GBD RR in the estimation of models or evaluate models against qualitative patterns in alcohol consumption.

### Disease probability models

We model the individual disease probability with a Hierarchical Bayes (HB) logistic regression, where the log odds of having the disease is a function of demographic, behavioral and other control (including socioeconomic, *BMI’*, *Region* and *Method*) variables. Working with observational data, causal effects can be estimated for counterfactual scenario evaluation if the model is correctly specified and includes all confounding covariates [24]. We assume that the probability of an individual receiving treatment (choosing certain behaviors in our case) is independent of the potential outcomes conditional on the confounding variables [25]. We specify model variables based on literature and use the same dimensions for heterogeneity of treatment effects (age and/or gender) as in the GBD meta-analysis results [e.g., 7, 26, 27].

*y*_*d*,*i*_ takes the value 1 if individual *i* has the non-communicable disease *d*, and 0 otherwise. It is a Bernoulli random variable with parameter *θ*_*d*,*i*_. We use the notation *a*[*i*] for the age bucket containing individual *i*, hence ${\alpha_{demog}}_{a[i],g[i]}$ refers to the parameter for *Age a* and *Gender g* interaction corresponding to the age and gender of individual *i*. We drop the disease index in the parameters below for brevity.

$$\begin{array}{l} logit\left(\theta_{IHD,i}\right)=\alpha_{demog}{}_{a\left[i\right],g\left[i\right]}+\beta_{BMI}{}_{a\left[i\right]}\;BMI_{i}^{\text{'}}+\;\alpha_{phys}{}_{a\left[i\right],m\left[i\right]}+\beta_{smoke}{}_{a\left[i\right].g\left[i\right]}Smoke_{i}\\ +\alpha_{alc}{}_{g\left[i\right],c\left[i\right]}+\alpha_{veg}{}_{v\left[i\right]}+\;\alpha_{fruit}{}_{f\left[i\right]}+\beta_{secsmoke}SecSmoke_{i}+\alpha_{trust}{}_{t\left[i\right]}+\beta_{edu}Education_{i}\\ +\beta_{inc}Income_{i}+\beta_{meth}Method_{i\;}+\alpha_{region}{}_{r\left[i\right]} \end{array}$$

$$\begin{array}{l} logit\left(\theta_{Diab,i}\right)=\alpha_{demog}{}_{a\left[i\right],g\left[i\right]}+\beta_{BMI}{}_{a\left[i\right]}\;BMI_{i}^{\text{'}}+\;\alpha_{phys}{}_{m\left[i\right]}+\beta_{smoke}{}_{g\left[i\right]}Smoke_{i}+\alpha_{alc}{}_{g\left[i\right],c\left[i\right]}\\ +\alpha_{veg}{}_{v\left[i\right]}+\;\alpha_{fruit}{}_{f\left[i\right]}+\beta_{secsmoke}SecSmoke_{i}+\alpha_{trust}{}_{t\left[i\right]}+\beta_{edu}Education_{i}+\beta_{inc}Income_{i}\\ +\beta_{meth}Method_{i\;}+\alpha_{region}{}_{r\left[i\right]} \end{array}$$

All parameters in the model are distributed normally with prior mean 0, indicating that the associated risk factor does not affect the disease probability, unless the data shows otherwise; and the hyper-prior distribution for the variance is an uninformative Inverse Gamma with parameters 5 and 50.

The proposed *Qualitative Informative HB* model does not include quantitative information in the prior distribution parameters, instead it restricts the relevant parameters based on the qualitative patterns listed in Table 1. For example, we expect the coefficient of smoker to be positive, and that for both the male and female gender, the RR is increasing with age group (see Table 1). Therefore, we impose the following constraints on the smoking coefficients in the IHD model:

$${\beta_{smoke}}_{a.g}\geq 0$$

$${\beta_{smoke}}_{a+1.g}\geq {\beta_{smoke}}_{a.g};for\;1\leq a\leq 6$$

These inequality constraints guide the estimation of the model parameters during the MCMC (Markov Chain Monte Carlo) simulation [28] and ensure that the parameters are in line with domain knowledge. The Bayes rule updates the prior distribution of model parameters with the observed data to yield their posterior distribution. In cases where the model has no closed-form solution, such as in the case of our HB models, the MCMC algorithm samples iteratively from approximate distributions and corrects them at each iteration to converge to the target, using Gibbs sampler and the Metropolis-Hastings algorithm. The Gibbs sampler draws each subset of parameters conditional on the value of all the others. Metropolis-Hastings algorithm implements an acceptance/ rejection rule such that if the new parameter estimate increases the posterior density then the parameter is updated, else the parameter is updated with the probability equal to the ratio of the new posterior density to the previous one [28].

We implement the hierarchical Bayes models with rJAGS tool [29] in the R environment. MCMC simulations are run with 3 chains of 5000 iterations each, excluding the 1000 burn-in iterations, and convergence is achieved.

### Disease probability model evaluation and benchmarks

Before we use the disease probability models in the simulator, we evaluate whether we are giving up on predictive accuracy as we insist on qualitative agreement with domain knowledge, by comparing the predictive accuracy of the proposed *Qualitative Informative* to the *Non-informative* HB and *Numerical Informative* HB as well as non-HB Logistic Regression models.

The benchmark *Non-informative* HB model does not guide or bias parameter estimation process with information from domain knowledge, it uses “non-informative priors”. The *Numerical Informative* HB model is the more traditional method of incorporating domain knowledge. The prior mean and variance of the relevant parameters are set according to the GBD RR results with natural log transformation, since the ln relative risk and odds ratio measures are approximately equal for low probability events [30].

To evaluate the proposed *Qualitative Informative* HB versus the benchmark models in terms of predictive accuracy we randomly partition the data into 70% train and 30% hold-out datasets stratified by survey year, and fit the models using the train data. The accuracy of predictions in the holdout data is evaluated with two measures:

1. For a well calibrated model, the predicted number of prevalent cases should be close to the actual number of prevalent cases. The expected number of prevalent cases is calculated by summing up the individual disease probabilities. Thousand samples from the joint posterior distribution of the model parameters provides the uncertainty intervals for the HB models. For the non-Bayesian benchmark models hundred bootstrap samples are used to train models and calculate predictions, which are similarly aggregated. We would like this interval to be small, reflecting confidence in the estimates and contain the actual value.
2. For a model that can correctly order individuals according to their disease probability, the Area Under the Curve (AUC) should be high. AUC is the probability that a randomly selected positive case will have a higher prediction score than a randomly selected negative case [31]. It is unaffected by calibration issues.

After confirming that *Qualitative Informative HB* is at least as accurate as the benchmark models, we train it using all the available data to use in the simulator. An inferior performance in terms of predictive accuracy would be an indication that the individual level data is not compatible with the qualitative patterns observed in the domain knowledge.

### DALY simulator

The DALY simulator calculates point and interval estimates for the % change in disease prevalence and DALYs for the given behavioral scenario *s*. The other inputs to the simulator are a) the Qualitative Informative HB disease probability model for each considered disease, i.e., (the joint posterior distribution of each model’s parameters), b) the individual level dataset for the targeted population—we use the 2016 data from the survey as the base case, reflecting the most recent demographics, behaviors, socioeconomics and control variable information available; and c) the disease, age and gender specific DALY-per- prevalent-case (DPP) estimates based on country level GBD results.

1. For a given scenario of behaviors, we first calculate the predicted probability of disease for each disease *d* and individual *i*, *θ*_*d*,*i*_|***x***_*i*,*s*_, where ***x***_*i*,*s*_ are the covariates containing the individual’s demographics, behaviors, socioeconomics and control variable information under scenario *s*.
2. Next, we calculate the expected DALYs due to each individual and disease under scenario *s*, *DALY*_*d*,*i*,*s*_, by multiplying each disease probability by the DALY-per-prevalent-case for that disease, *DPP*_*d*,*a*[*i*],*g*[*i*]_ for the age and gender of the individual *i*.
  $$DALY_{d,i,s}={\theta_{d,i}|x}_{i,s}*DPP_{d,\;a[i],g[i]}$$
3. Since the survey is a representative sample of the population, the population level expected DALYs for the given scenario is the sum of the individual expected DALY values, ∑_*i*_ *DALY*_*d*,*i*,*s*_, except for a multiplier to represent the sampling rate and the potential self-reporting bias in the survey. We only report the %change in the disease prevalence and DALYs which are unaffected by this unknown multiplier.
  The %change in the population level disease DALYs due to the behavior change as specified in the scenario compared to the base case is calculated as follows.
  $$\%\Delta DALY_{d,s}=\left(\sum_{i}DALY_{d,i,s}-\sum_{i}DALY_{d,i,base}\right)/\sum_{i}DALY_{d,i,base}$$
  The %change in disease prevalence is calculated similarly for each disease, by aggregating the predicted disease probability values under base case and scenario behaviors.
  $${\%\Delta prevalence}_{d,s}=(\sum_{i}{\theta_{d,i}|x}_{i,s}-\sum_{i}{\theta_{d,i}|x}_{i,base})/\sum_{i}{\theta_{d,i}|x}_{i,base}$$
4. In order to properly propagate the uncertainty in the disease probability model estimates, we repeat steps 1–3 for a thousand samples of the joint posterior distribution of the disease model parameters and report the mean, and the 5^th^ and 95^th^ percentiles as the 90% uncertainty interval [24].

### Behavior change scenarios

The first objective in the 2019–2023 Strategic Plan of the Turkish Ministry of Health is to promote healthy lifestyle with the following top three goals of developing healthy eating habits, developing physically active lifestyle habits, and reducing tobacco consumption in the population (http://www.sp.gov.tr/tr/stratejik-plan/s/1652/Saglik+Bakanligi+2019-2023). The ministry has a comprehensive set of measures, including educational, cultural and tactical programs to address these objectives. Turkey has an ongoing battle to reduce smoking and second-hand smoke with multiple initiatives, and is facing a growing obesity challenge–in our data we also see a steady increase in the average BMI over the years. the Our goal in this exercise is to provide quantitative measures for the potential to reduce the amount of burden by improving behaviors in these categories, rather than projecting the impact of specific interventions. Hence we evaluate scenarios that involve wholesale change in behavior patterns in physical activity, smoking and second-hand smoking, and fruit and vegetable consumption.

As mentioned earlier, we use the 2016 data from the survey as the base case, reflecting the most recent demographics, behaviors, socioeconomics and control variable information available. The marginal distribution of the behaviors in the base case is provided in Table 2. We observe that Smokers are the largest group at the most unfavorable behavior level, with 44% of the adult population, whereas the majority of the population (53% and 62% respectively) is consuming fruits and vegetables at the highest level. There is a moderate correlation between fruit and vegetable consumption (0.49), and some positive correlation between smoking and exposure to second-hand smoke (0.14).

**Table 2:** The marginal distribution of the behaviors involved in the scenarios for the base case 2016 survey results.

Behavior	% population
**Physical Activity**	
Inactive	18%
Insufficiently Active	45%
Active	10%
Highly Active	27%
**Smoking**	
Non-smokers	56%
Smokers	44%
**Second-hand smoke**	
Non-exposed	74%
Exposed	26%
Vegetable consumption	
None	1%
<once a week	3%
1–3 times a week	16%
4–6 times a week	19%
One or more per day	62%
Fruit consumption	
None	2%
<once a week	6%
1–3 times a week	23%
4–6 times a week	17%
One or more per day	53%

We evaluate the following potential behavior change scenarios. 1) Increase Physical Activity: All individuals with known activity level move to the next higher activity level (“Inactive” -> “Insufficiently Active” -> “Active” -> “Highly Active”). 2) Eliminate Smoking and Secondhand Smoking: All individuals move to “Non-smoking” and “Un-exposed to Secondhand Smoking”. 3) Increase Fruit and Vegetable Consumption: All individuals move to the next consumption level (“None” -> “Less than once a week” -> “1–3 times a week” -> “4–6 times a week” -> “One or more per day”). There is no change for individuals who are already at the most desirable level for that behavior. As explained earlier, since the alcohol consumption in the population is very low, we do not investigate scenarios involving reducing alcohol consumption. Our motivation for moving each individual to the next risk category rather than to the “optimal” level is practical. We think, for example, that an inactive person is more likely to increase activity in response to an intervention and reach Insufficiently Active level than Highly Active level.

## Results

### Evaluation of disease probability models

Table 3 summarizes whether the estimated parameters of each benchmark disease probability model are in agreement with the qualitative patterns observed from the meta-analysis results that were described in Table 1. For example, the first line indicates that the simple logistic regression has parameters for the effect of physical activity on IHD with the “wrong” sign; in this case indicating physical activity is associated with higher IHD risk at least for some groups. We observe that informing the model with prior information is needed to get the sign of the parameters correctly, as in the Qualitative Informative and Numerical Informative HB models vs the Non-Informative HB and Logistic Regression. But the traditional Numerical Informative HB is not able to provide the expected monotonicity of the effects as seen in the lower half of the table.

**Table 3:** Evaluation results for the disease probability models—Agreement with the expected qualitative patterns.

Disease probability models by disease	Proposed		Benchmark models					
	Qualitative Informative HB		Non-Informative HB		Numerical Informative HB		Logistic regression	
	IHD	Diabetes	IHD	Diabetes	IHD	Diabetes	IHD	Diabetes
**Sign of the Effect**								
Physical Activity	✓	✓	×	✓	✓	✓	×	✓
Smoking	✓	✓	✓	✓	✓	✓	✓	✓
Second-Hand Smoking	✓	✓	✓	×	✓	✓	✓	×
BMI	✓	✓	✓	✓	✓	✓	✓	✓
**Shape of Effect**								
Male with Age	✓	✓	×	×	✓	×	×	×
Female with Age	✓	✓	✓	×	✓	×	✓	×
Physical Activity with Level	✓	✓	×	✓	×	✓	×	✓
Physical Activity with Age	✓	NA	×	NA	×	NA	×	NA
Male Smoking with Age	✓	NA	×	NA	×	NA	×	NA
Female Smoking with Age	✓	NA	×	NA	✓	NA	×	NA
BMI with Age	✓	✓	×	×	✓	×	×	×

**Fig 2:**
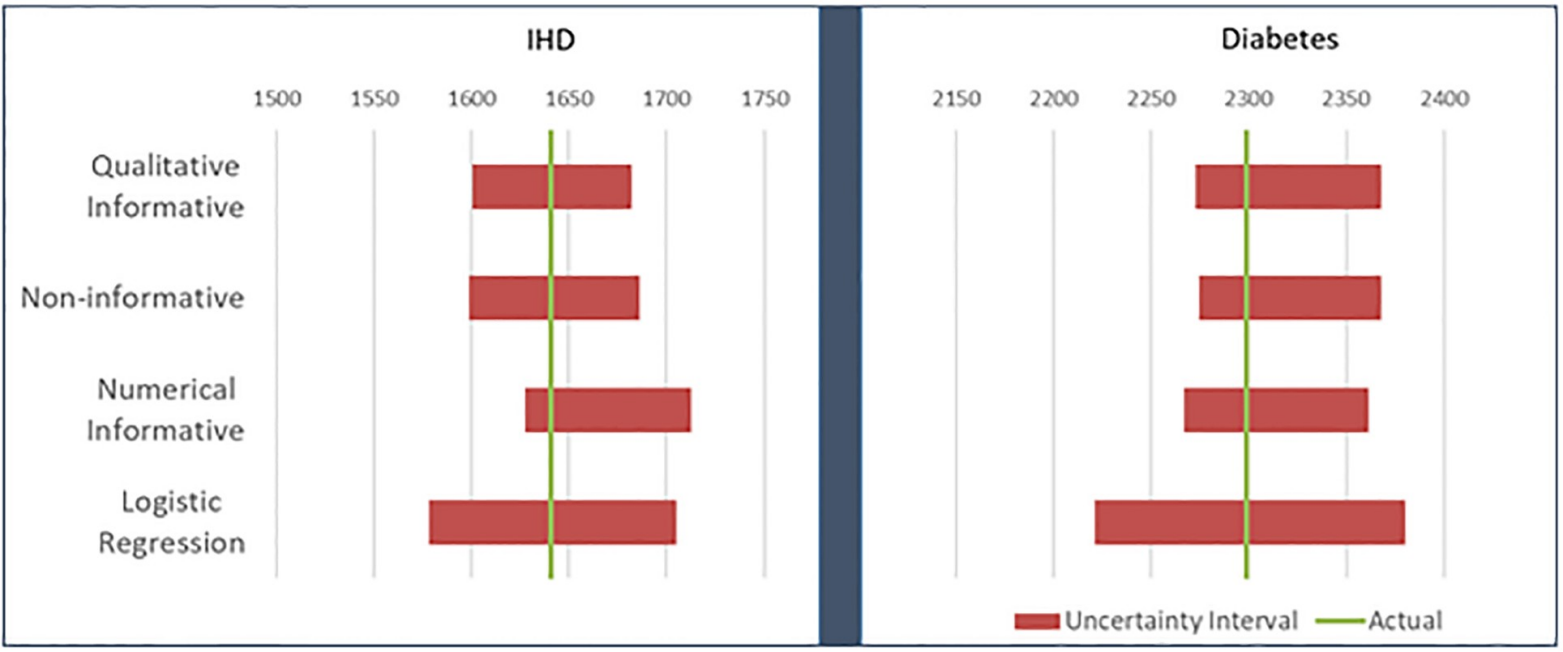
Predicted number of prevalent cases in the holdout population with 90% uncertainty interval.

Hence, the Qualitative Informative HB is the only model that complies with all the listed criteria in for all diseases, thanks to the constrained prior distributions on its parameters. Note that these constraints do not decrease its predictive accuracy. As seen in Fig 2, the uncertainty interval for the prediction of number of prevalent cases in the holdout population for the proposed Qualitative Informative HB contains the actual value and it is considerably shorter than the non-HB Logistic Regression. Similarly, its uncertainty intervals are adequate for all covariate subpopulations (not shown).

**Fig 3:**
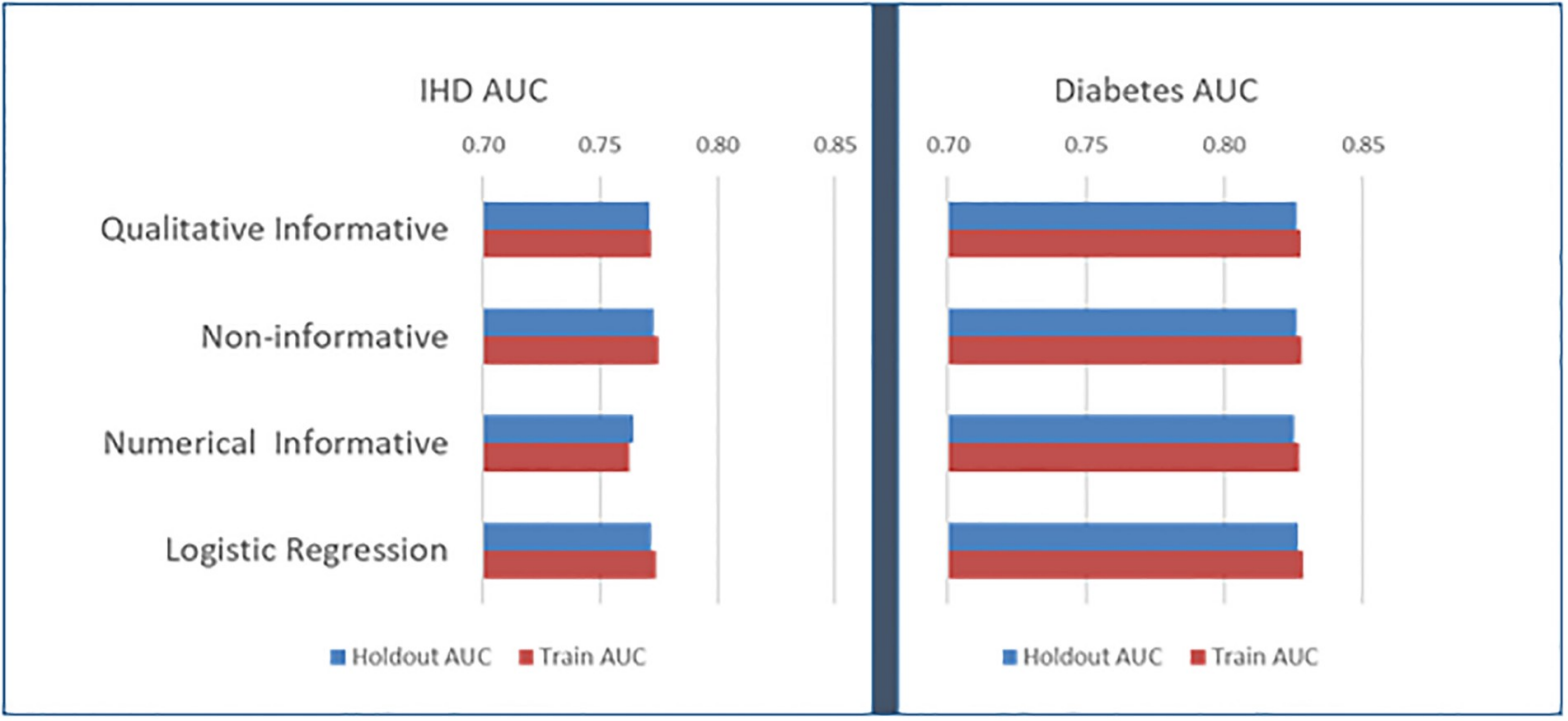
Predictive accuracy of disease probability, measured in holdout AUC.

As seen in Fig 3, the holdout AUC of the Qualitative Informative HB is consistently among the best for each disease model. Overall, its holdout AUC ranges from 0.77 to 0.83, which is adequate. The models do not exhibit overfitting, as the train accuracy is very close to the holdout accuracy.

As a result of our evaluation and comparison with the benchmark models, we conclude that the Informative Qualitative HB disease probability models provide are the only ones to agree with the expected qualitative patterns of relative risks and provide the best predictive accuracy.

### Impact of behaviors on disease probability and related discussion

**Fig 4:**
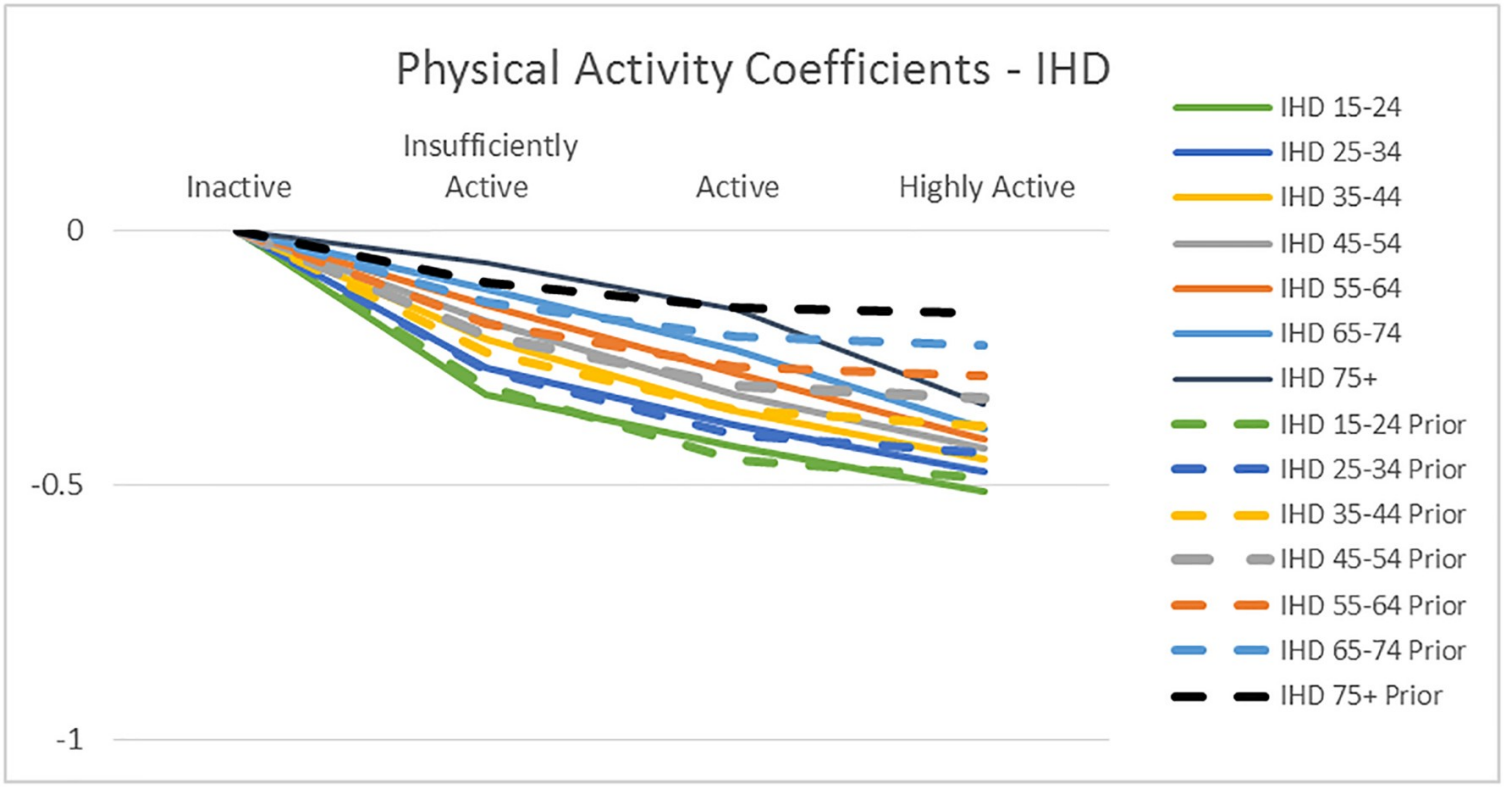
The mean physical activity coefficients of the IHD disease probability model by age group, compared with the values implied by GBD meta-analysis RR (prior).

**Fig 5:**
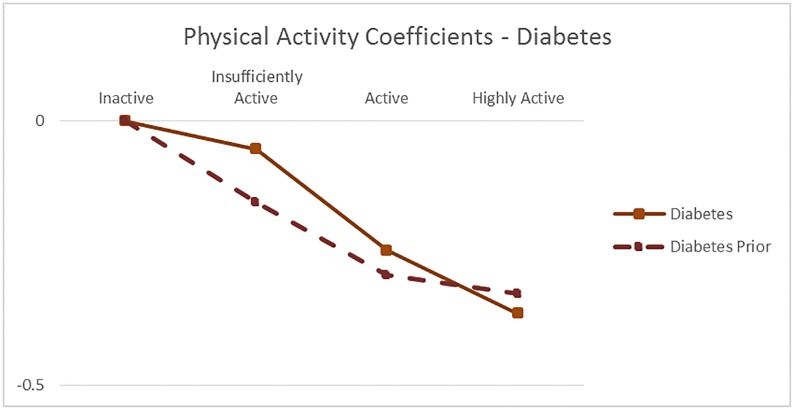
The mean physical activity coefficients of the diabetes disease probability model, compared with the values implied by GBD meta-analysis RR (prior).

Fig 4 through Fig 8 exhibit the mean model coefficients of the Informative Qualitative HB models associated with the behavioral risk factors investigated in the scenarios, along with the values implied by the GBD meta-analysis results, where available. The model coefficients can be interpreted as the natural log of the relative risk for the behavior compared to its reference level, controlling for other behaviors included in the model (smoking, second hand smoking, physical activity, fruit and vegetable consumption, number of trusted individuals and alcohol consumption), demographics (age and gender), socioeconomics (education and income), BMI, region, and survey controls. While the range of the y-axis range differs among figures, the gridlines in all graphs are set 0.5 units apart, Figs 4 and 5 illustrate the impact of *Physical Activity* on IHD and Diabetes disease probability, respectively by age group. For example, the solid green line in Fig 4 shows the Physical Activity coefficients of the IHD model for the 15–24 year old group, i.e., ${\alpha_{phys}}_{1,m}$, indicating that the risk of IHD decreases with more Physical Activity, and that the Highly Active individuals have roughly 62% (e^-0.48^) of the risk of the Inactive in the same age group–other things being equal. The dotted green lines on the same graph show the equivalent values implied by the GBD meta-analysis results.

**Fig 6:**
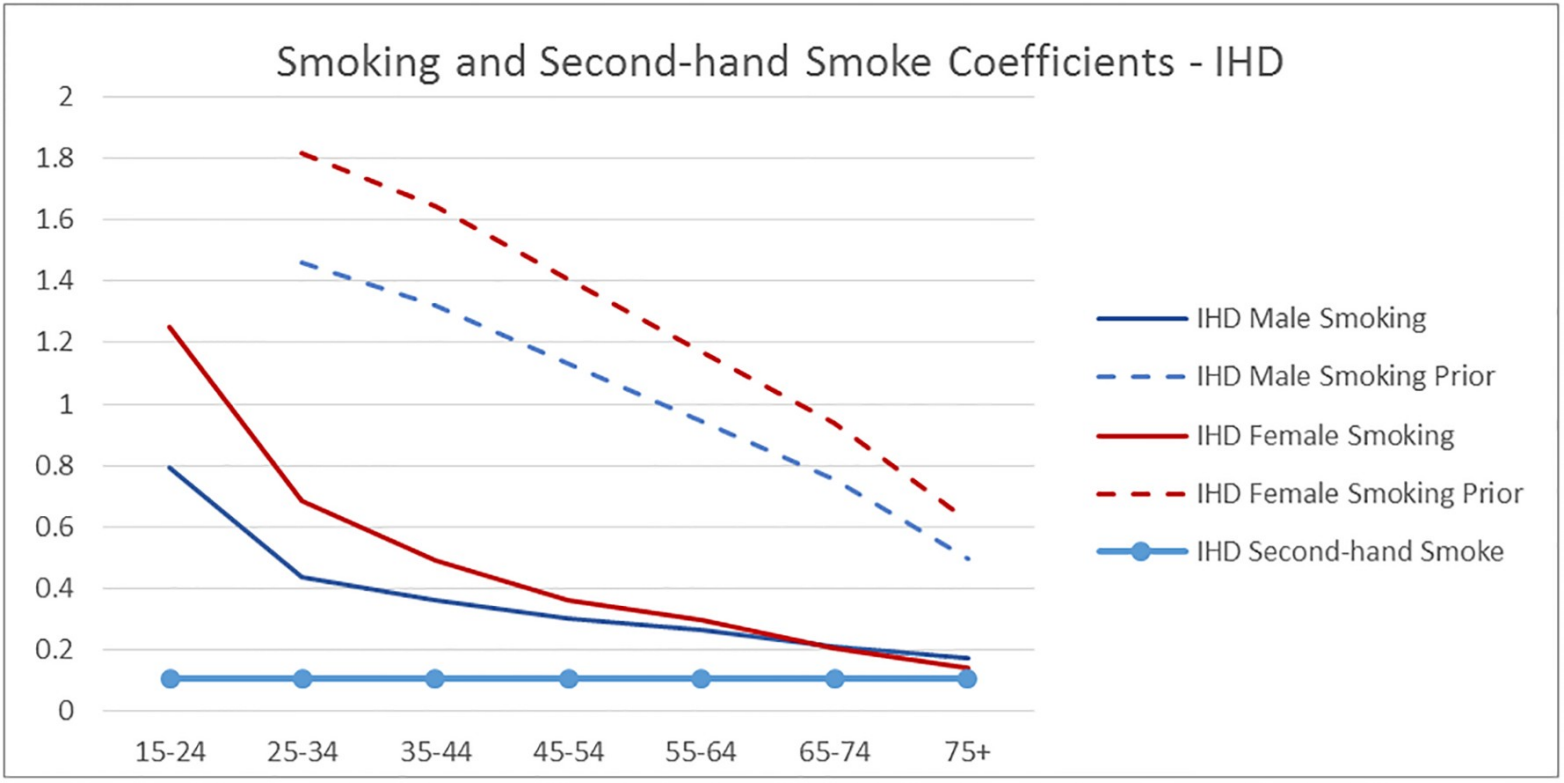
The mean smoking coefficients of the IHD disease probability models by age group, compared with the values implied by GBD meta-analysis RR (prior)—No GBD values available for second-hand smoke.

**Fig 7:**
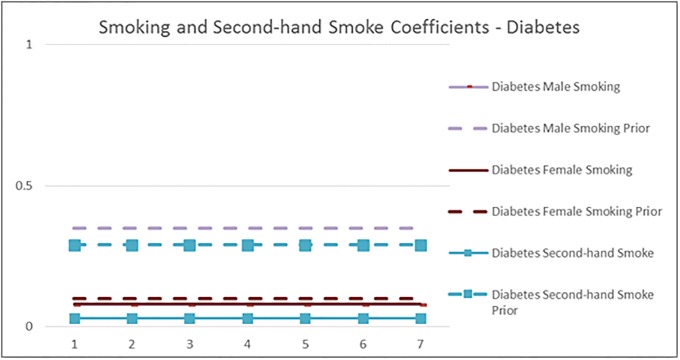
The mean smoking and second-hand smoking coefficients of the diabetes disease probability model by age group, compared with the values implied by the GBD meta-analysis (prior). Male and Female smoking lines overlap in the graph.

Figs 6 and 7 show the impact of Smoking and Second-hand smoke on IHD and Diabetes, respectively. For example, the solid red line in Fig 7 shows that the IHD risk of female smokers relative to female non-smokers is positive for all age groups and goes down from 1.25 (which corresponds to RR of 3.5) for the 15–24 age group to 0.14 (1.15 RR) for the 75+ age group.

We can see that in all graphs the qualitative patterns are maintained: RR of disease decreases with physical activity, smoking and second hand smoking increase disease risk. The RR decreases with age unless it is assumed to be constant. But there are quantitative differences: The impact of *Smoking* on all diseases is substantially lower than the GBD estimates. Potential reasons for the discrepancy include local sensitivities being different than global, the estimation controlling for other behaviors and socioeconomics in addition to age and gender, and that the GBD smoking measure is for 5 year lagged smoking, compared to the use in the last year in our dataset. In fact, the proposed model coefficients are well in line with estimates from a newer meta-analysis based on 141 cohort studies [32]: They report pooled relative risk estimates 1.48 for 1 cigarette per day, and 2.04 for 20 cigarettes per day for men, and 1.57 and 2.84 for women (implied coefficients of 0.39 and 0.71 for men, and 0.45 and 1.04 for women), pointing out that the RR decreases with age. Another difference we observe is that the IHD risk of the *Highly Active* in older age groups continues to reduce with more intense activity beyond Active, unlike GBD. The results from a prospective cohort analysis corroborates this pattern: They find that in older adults (above and below 75 years), greater physical activity, in terms of time, intensity and distance, is inversely associated with the risk of coronary heart disease [33].

**Fig 8:**
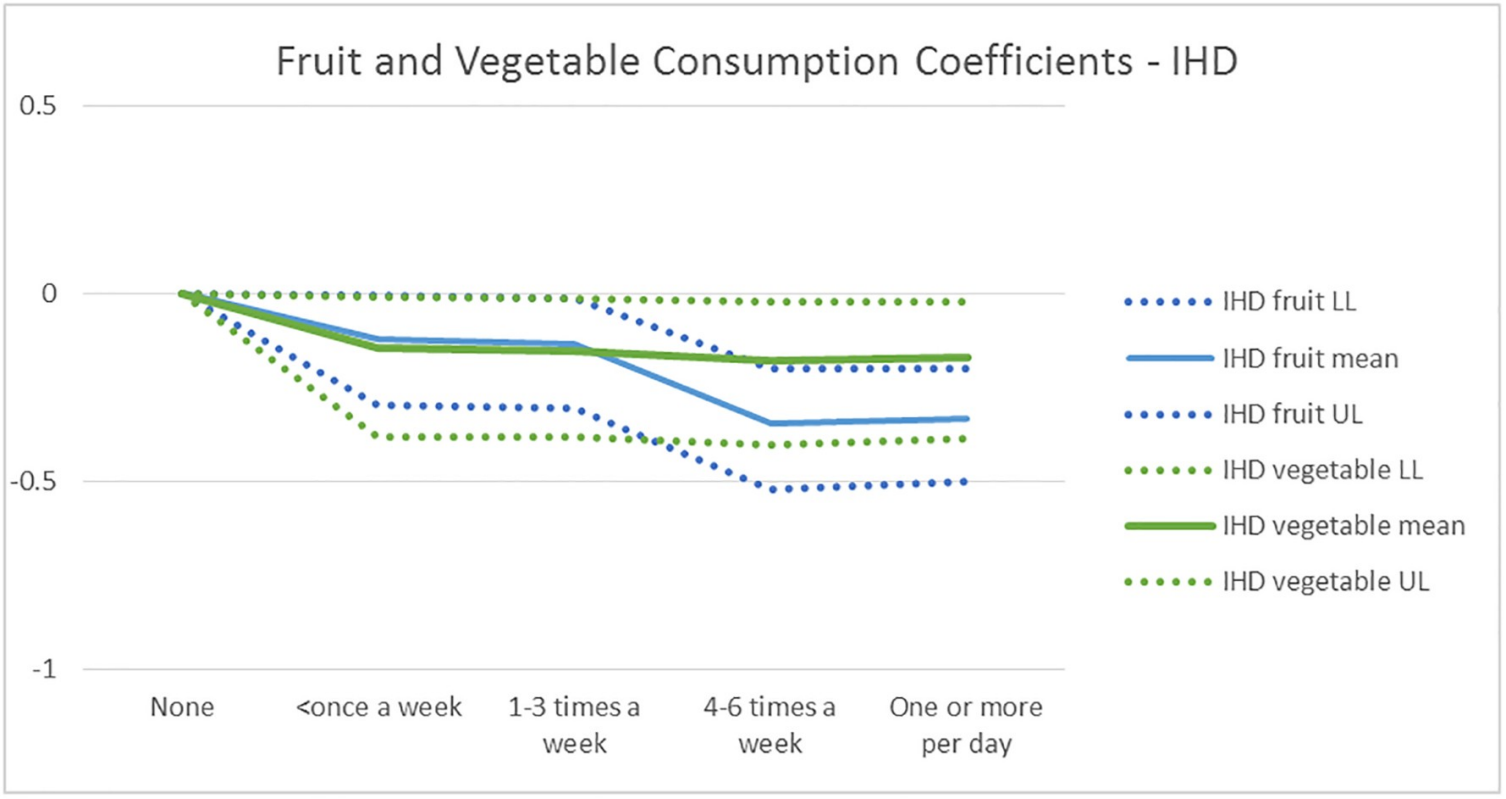
The mean and 95% uncertainty interval for the fruit and vegetable consumption coefficients for the IHD disease probability model. The GBD RR estimate is only available for low consumption of fruits and vegetables (<100 grams/day), and implies that both increase the risk of IHD.

**Fig 9:**
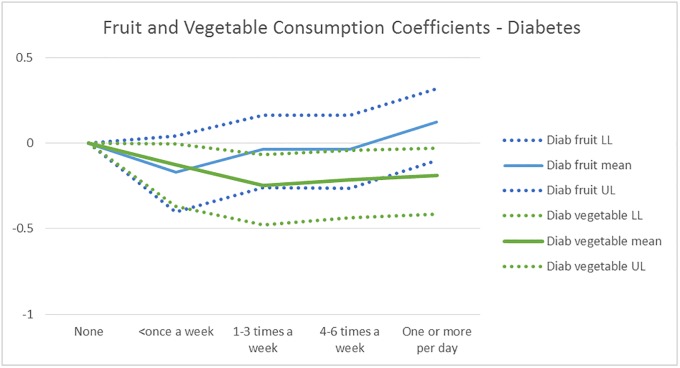
The mean and 95% uncertainty interval for the fruit and vegetable consumption coefficients for the diabetes disease probability model. The GBD RR estimate is available for low consumption of fruits (<100 grams/day) only and implies higher risk of diabetes.

Figs 8 and 9 show the mean and the 95% credible interval of the posterior distribution for the Fruit and Vegetable consumption coefficients in the IHD and Diabetes models, respectively. GBD RR estimates indicate that low consumption (<100 grams/day) fruits and vegetables increases IHD probability, which is supported by Fig 8. Further, Fig 8 suggests IHD risk goes down with increased fruit and vegetable consumption, vegetable consumption benefit stabilizing at1-3 times a week, while fruit consumption provides additional benefits until 4–6 times a week. While the measure of consumption is different, the findings that the cardiovascular risk reduces with fruit and vegetable consumption up to a threshold, with a larger effect for fruit consumption, are also reported in a meta-analysis of prospective cohort studies [27] The relative risk they report at the threshold corresponds to -0.20 for vegetable and -0.26 for fruit consumption, which are similar to our estimates (and uncertainty intervals) -0.17 (-0.39–0.02) and -0.33 (-0.50–0.20), respectively.

Fig 9 suggests that increased consumption of vegetables decreases Diabetes risk–up to a threshold -, while fruit consumption does not. The uncertainty interval of the fruit consumption coefficients contains 0 at all consumption levels, and even suggests higher Diabetes risk than no consumption at the highest level of consumption. While there are many studies finding that fruit and vegetable consumption together reduce the Diabetes risk, e.g. [34, 35], there is conflicting evidence on the effect of fruit consumption, which is partly due to measurement difficulties including usage of different units, and correlation of fruit and vegetable consumption [35]. A study trying to separate the impact of fruit versus vegetable consumption finds that vegetable but not fruit consumption reduces the risk of type 2 Diabetes [36]. Another one finds that only green leafy vegetables reduce the Diabetes risk, while fruit or vegetable consumption separately do not [37]. Given this set of evidence, we zero out the fruit consumption effect on Diabetes for scenario evaluation purposes.

### Scenario impact on DALYs

**Fig 10:**
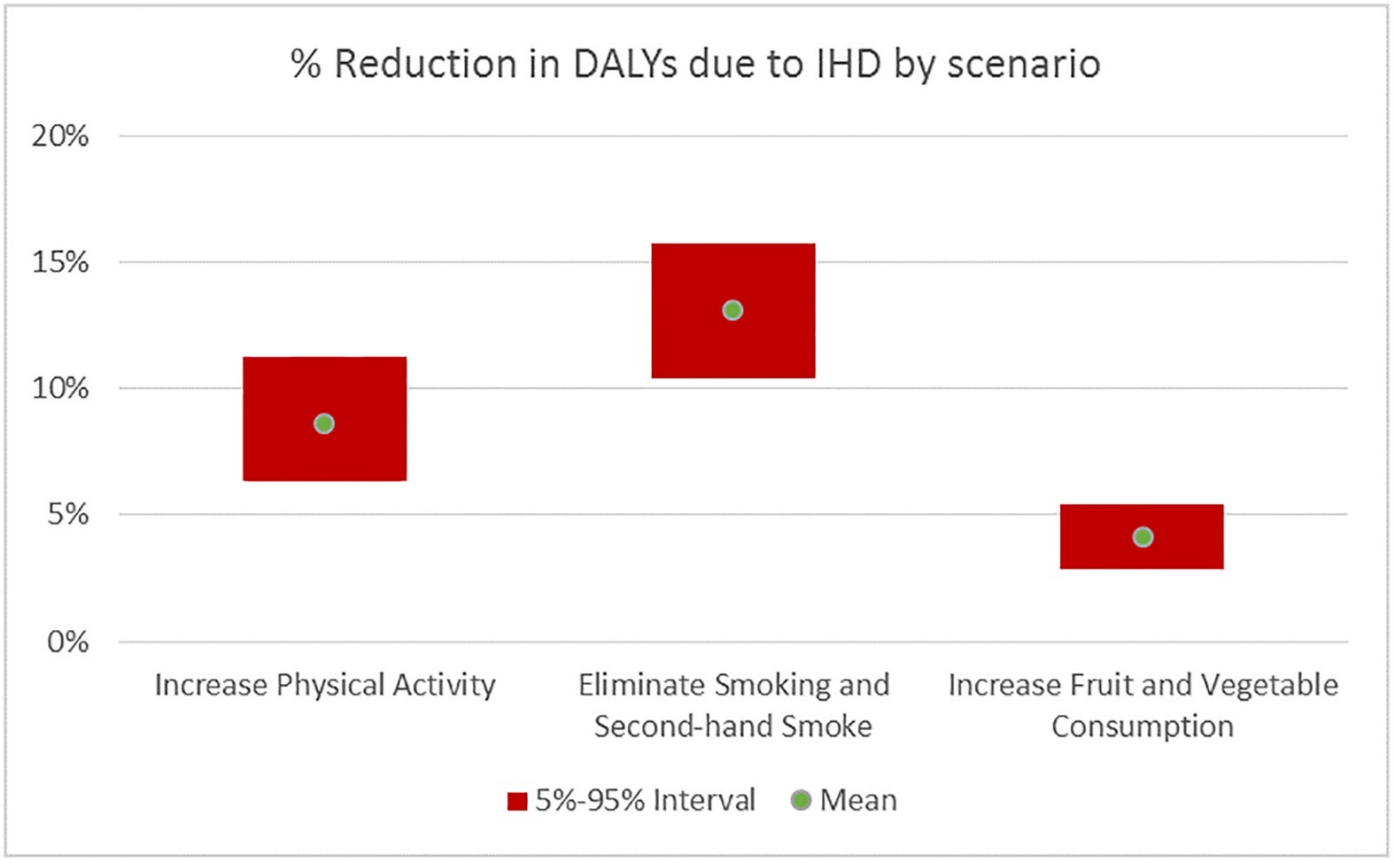
Reduction in the disease burden of IHD by scenario. Mean and 90% uncertainty interval of %Reduction in IHD DALYs due to change in behavior.

**Fig 11:**
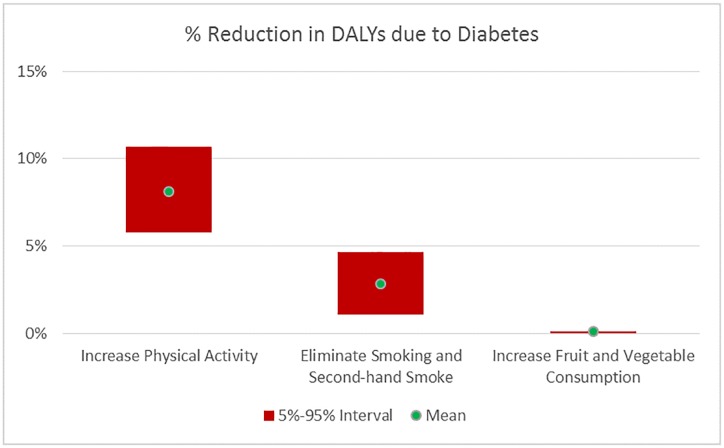
Reduction in the disease burden of diabetes by scenario. Mean and 90% uncertainty interval of %Reduction in diabetes DALYs due to change in behavior.

Figs 10 and 11 illustrate the mean and 90% uncertainty interval for the expected % reduction at the population level in the IHD and Diabetes DALYs, respectively, due to each scenario. Table 4 provides these numbers as well as the % reduction in the prevalence of each disease with the mean and 90% uncertainty interval.

**Table 4:** Mean and 90% uncertainty interval estimates (in parentheses) for the expected % reduction in DALYs and prevalence due to each disease by scenario.

Scenario	Increase Physical Activity	Eliminate Smoking & Secondhand Smoking	Increase Fruit & Vegetable Consumption
Disease DALY reduction			
IHD DALY	8.6 (6.4 11.2)	13.1 (10.4 15.8)	4.1 (2.8 5.4)
Diabetes DALY	8.1 (5.8 10.6)	2.8 (1.1 4.6)	0.1 (0 0.1)
Disease prevalence reduction			
IHD prevalence	8.7 (6.8 10.9)	13.4 (11.1 15.6)	4.3 (3.2 5.4)
Diabetes prevalence	8.3 (6.1 10.4)	2.9 (1.4 4.5)	0.1 (0.0 0.1)

Eliminating Smoking and Secondhand Smoke has the highest potential to reduce the IHD DALYs, followed by Increasing Physical Activity with mean reductions of 13.1% and 8.6% respectively. There is some overlap in their 90% uncertainty intervals, indicating that their impact is not significantly different, while the Increasing Fruit and Vegetable Consumption scenario has a clearly lower impact with mean reduction of 2.1%. In terms of % reduction in Diabetes DALYs, Increasing Physical Activity has higher impact than Eliminating Smoking and Secondhand Smoke, which has higher impact than the Increasing Fruit and Vegetable Consumption scenario.

Increasing Physical Activity reduces the prevalence of both diseases by similar amounts (8.7% and 8.3% for IHD and Diabetes respectively), while Eliminating Smoking and Secondhand Smoking predominantly decreases IHD prevalence (13.4%) and to a smaller degree Diabetes prevalence (2.9%)–see Table 4.

Increasing Fruit and Vegetable Consumption, on the other hand, reduces IHD prevalence in the population by 4.3% while not affecting the Diabetes prevalence. An important driver of this result is the high level of fruit and vegetable consumption in the population, with 53% (62%) at the maximum levels respectively, as seen in Table 2. In terms of IHD, 80% of the population is at or beyond maximum benefit levels for fruit or vegetable consumption. Only 4% of the population stands to decrease their Diabetes risk by increasing their vegetable consumption, as fruit consumption does not decrease Diabetes risk based on the analysis results.

Table 5 provides the %DALYs of IHD and Diabetes attributed to exposure to the risk factors in Turkey by the GBD 2016 study, which quantifies the %change in the burden that would result from moving all adults to the theoretical minimum risk level (TMREL) using estimates of exposure and the global RR. The Tobacco impact estimates include Smoking and Second-hand smoke, and are more than two to six times higher than the local DALY impact estimates for IHD and for Diabetes. The Increase Physical Activity scenario is not the same as the one implied by the GBD Low Physical Activity risk attribution which involves moving all adults to the TMREL 3000–4500 MET min per week [7], versus the next level of activity. Nevertheless, we do not observe a systematic bias for physical activity: GBD attributable burden estimates are lower than the local impact estimates for Diabetes and substantially higher for IHD with a wide uncertainty interval. The GBD study assumes TMREL for fruit and vegetable consumption as 200 to 300 grams, and 290 to 430 grams per day, respectively. The burden attributable to Low Fruit and Vegetable Consumption are higher than the local estimates; particularly in the case of Diabetes where the local analysis suggests almost no impact.

**Table 5:** Mean and uncertainty interval estimates for the % disease DALYs in Turkey attributable to each risk factor by disease. Source GBD 2016 (https://vizhub.healthdata.org/gbd-compare/).

Risk factor	Low Physical Activity	Tobacco	Low Fruit and Vegetable Consumption
Attributable burden as % of disease DALYs			
IHD DALY reduction	11.1 (5.3 18.4)	35.4 (33.5 37.3)	6.2 (1.3 15.0)
Diabetes DALY reduction	3.4 (0.7 6.4)	18.4 (13.3 23.0)	4.3 (0.6 9.5)

## Discussion

The introduced method provides point estimates and uncertainty intervals for the national impact of behavioral scenarios on disease burden and prevalence that are based on national relative risk and local joint distribution of behaviors, demographics, socioeconomics and other controls. As seen in the case study, while the estimated relative risks are in line with the global patterns, the impact estimates are quantitatively and qualitatively different than the GBD attributable burden estimates. The method also allows policy makers to evaluate practical behavior scenarios without double counting the effect due to changes in multiple behaviors.

An implication of the study for health policy makers in Turkey is that while battling smoking provides a larger benefit against the largest cause of disease burden of the country, i.e.; IHD, encouraging physical activity results in very substantial benefits against IHD as well as Diabetes burden, which has been growing at an alarming pace [38]. Promoting consumption of fruit and vegetables has limited potential benefits, due to the high level of fruit and vegetable consumption in the population and lack of impact for decreasing the Diabetes burden.

The proposed methodology can be used in other contexts to evaluate the local impact of behavioral scenarios on disease prevalence and DALYs, where sizable individual level observational data is available. It can also be extended to combining the DALYs due to multiple diseases without double counting, provided that the self-reporting biases can be measured for each disease. Further, the method can be extended to facilitate optimal targeting of subpopulations to maximize the benefit expected from behavioral public health scenarios.

Limitations of the study include that the data is based on self-reported outcomes and that current behaviors that may or may not reflect the historical behavior patterns. The disease probability models assume that having the disease does not impede the behaviors whose impact on the disease are investigated, and should be interpreted accordingly to rule out reverse-causality. Lastly, despite the large sample size and the regularization effect of the sign and shape constraints, residual confounding effects cannot be ruled out in observational studies.

## Supporting information

S1 Data (XLSX)

S2 Data (TXT)
